# Polyphenolic Composition of Lentil Roots in Response to Infection by *Aphanomyces euteiches*

**DOI:** 10.3389/fpls.2018.01131

**Published:** 2018-08-03

**Authors:** Navid Bazghaleh, Pratibha Prashar, Randy W. Purves, Albert Vandenberg

**Affiliations:** Department of Plant Sciences, University of Saskatchewan, Saskatoon, SK, Canada

**Keywords:** lentil, *Lens ervoides*, *Lens culinaris*, polyphenols, *Aphanomyces euteiches*, root rot, liquid chromatography, mass spectrometry

## Abstract

Polyphenols comprise the largest group of plant secondary metabolites and have critical roles in plant physiology and response to the biotic and abiotic environment. Changes in the content of polyphenols in the root extracts and root tissues of wild (*Lens ervoides*) and cultivated (*Lens culinaris*) lentil genotypes were examined in response to infection by *Aphanomyces euteiches* using liquid chromatography mass spectrometry (LC-MS). Genotype, infection and their interaction determined the composition of polyphenols in lentil roots. The levels of several polyphenols were lower in the root extract of the low-tannin genotype *L. culinaris* ZT-4 compared to *L. ervoides* L01-827A. Kaempferol derivatives including kaempferol dirutinoside and kaempferol 3-robinoside 7-rhamnoside were more concentrated in the healthy root tissues of *L. ervoides* L01-827A than in *L. culinaris* genotypes. Infection increased the concentration of kaempferol, apigenin, and naringenin in the root tissues of all genotypes, but had no effect on some polyphenols in the low-tannin genotype *L. culinaris* ZT-4. The concentrations of apigenin, naringenin, apigenin 4-glucoside, naringenin7-rutinoside, diosmetin, and hesperetin 7-rutinoside were higher in the infected root tissues of *L. ervoides* L01-827A compared with the *L. culinaris* genotypes. Organic acids including coumaric acid, vanillic acid, 4-aminosalicylic acid, 4-hydroxybenzoic acid, and 3,4-dihydroxybenzoic acid effectively suppressed the *in-vitro* hyphal growth of *A. euteiches*. Some of these bioactive polyphenols were more concentrated in roots of *L. ervoides* L01-827A but were low to undetectable in ZT-4. This study shows that genotypic differences exist in the composition of root polyphenols in lentil, and is related to the response to infection caused by *A. euteiches*. Polyphenols, particularly the organic acid content could be useful for selection and breeding of lentil genotypes that are resistant to Aphanomyces root rot (ARR) disease.

## Introduction

Lentil (*Lens culinaris* Medik.) is an important cool season legume, grown in more than 70 countries around the world. Canada is the largest lentil producer, contributing nearly 41% of the global production (FAO, 2017). Lentil seeds are a rich source of proteins, carbohydrates, vitamins, minerals, fibers and antioxidants (Roy et al., 2010). They also contain non-nutritional compounds such as tannins and phytic acid that serve as a defense mechanism against pathogens, insects and parasites (Urbano et al., 2007; Constabel et al., 2014; Sánchez-Chino et al., 2015). *Lens* species differ in the size of seeds (5–90 mg per seed) and the color and pattern of seed coats, which vary from yellow or gray to dark brown (Ladizinsky, 1979a,b; Ferguson et al., 2000). As a legume crop, lentil provides agro-ecological services by incorporating atmospheric nitrogen into the soil through biological fixation, and promoting the diversity of soil microbial communities (Borrell et al., 2016).

*Aphanomyces euteiches* is a soil-borne oomycete that causes Aphanomyces root rot (ARR) in many legume crops such as lentil, pea (*Pisum sativum* L.), alfalfa (*Medicago sativa* L.), common bean (*Phaseolus vulgaris* L.), and red clover (*Trifolium pratense*; Wicker et al., 2001; Chatterton et al., 2016). This pathogen has recently been reported in western Canada (Armstrong-Cho et al., 2014; Chatterton et al., 2016). ARR can cause major crop losses especially under environmental conditions that are conducive to pathogen growth and disease development (McPhee, 2003; Gossen et al., 2016). Susceptible crop plants generally have a limited range of genetic resistance. No effective seed treatment is available for protection against *A. euteiches*, and its spores are long-lived in soil (Gossen et al., 2016). These characteristics make ARR the most difficult and serious root pathogen of susceptible legumes. Crop rotation and the use of cultivars with partial resistance are the only efficient ways to control ARR (Wicker et al., 2001; Moussart et al., 2013).

Polyphenols are the largest group of secondary metabolites and have very diverse structures (Cheynier, 2012) that make them unique and multifunctional natural products in plants (Quideau et al., 2011). They often have important ecological roles and are involved in a range of functions in plant growth, development, and defense. Polyphenols can attract, repel or protect plants against insects, fungi, bacteria, and viruses (Bennett and Wallsgrove, 1994; Daayf et al., 2012; Olivoto et al., 2017). Synthesis of polyphenols is a complex process that is associated with the shikimate, malonate, and phenylpropanoid pathways (Lattanzio, 2013) and is encoded by multiple genes (Vermerris and Nicholson, 2008; Olivoto et al., 2017). A detailed understanding of how these polyphenols fit into the biochemical pathways for disease resistance is necessary to develop strategies to control plant diseases.

The roots of plants are able to produce a diversity of compounds that directly or indirectly influence microbial species (Lanoue et al., 2010; Baetz and Martinoia, 2014; Bazghaleh et al., 2016). The production of antimicrobial polyphenols have been reported in various plant species (Puupponen-Pimiä et al., 2001; Deng et al., 2015; Shalaby and Horwitz, 2015; Pagliarulo et al., 2016) which could be independent or part of a plant's response to infection. Some polyphenols accumulate in plant tissues even prior to an active defense response (Nicholson and Hammerschmidt, 1992; Balmer et al., 2015). Knowledge of the components of the range of polyphenols in the root tissues and root extracts of crop plants and the potential effect of those compounds on pathogenic microbial species could be used as a first step for identifying plant genotypes that are more resistant to plant diseases (Wink, 1988; Reuveni et al., 1991; Wille et al., 2018).

Polyphenols have been detected in plants roots and their effects on soil pathogens described (Evidente et al., 2010), however, little is known about the composition of these metabolites, particularly in legume plants. This study tested the hypothesis that lentil genotypes have different compositions of polyphenols in their root tissues and root extracts. We also hypothesized that infection by *A. euteiches* alters the accumulation of specific polyphenols in the root tissues of lentil, and that some of these might inhibit the hyphal growth of *A. euteiches*. We compared the polyphenol profiles found in root tissues and root extracts of selected genotypes of cultivated (*L. culinaris*) and wild (*Lens ervoides*) lentil, including a low-tannin cultivar, using liquid chromatography-mass spectrometry. The effect of the individual polyphenols on the growth of *A. euteiches* was investigated *in vitro*. The aim of this study was to identify potential polyphenols that could be involved in providing resistance to ARR in lentil.

## Materials and methods

### Composition of polyphenols in root extracts of lentil

#### Experimental design and plant growth conditions

Seeds of the *L. culinaris* cultivars ZT-4 (low-tannin), CDC Maxim (gray seed coat), Eston (green seed coat), and *L. ervoides* genotype L01-827A (wild type seed coat) were obtained from the Crop Development Centre at the University of Saskatchewan (Saskatoon, SK, Canada). The lentil genotypes were selected based on differences in the color and polyphenolic profile of their seed coats (Mirali et al., 2016a,b, 2017). The seeds were scarified by puncturing the seed coats using a small blade. The seeds were surface sterilized by successive immersion in 95% ethanol for 30 s, in sterile distilled water for 30 s, in 2.5% Javex® bleach solution (sodium hypochlorite) for 2 min, and then in distilled water for 5 min. The seeds were germinated on moist, sterile filter paper in Petri dishes in the dark at 25°C for 72 h prior to use. Eight germinated seeds of each genotype were transplanted into a 10 cm plastic pot filled with porous ceramic media (Profile® Greens Grade™, BrettYoung, Edmonton, AB). The planting holes in the media were treated with a peat-based *Rhizobium leguminosarum* inoculant (Nitragin Nitrastick GC®, Nitragin Inc., Brookfield, WI, USA). The pots were arranged as a randomized complete block design in four replicates. Plants were grown under 22/16°C d/night temperatures and 16/8 h d/night length for 30 d in a growth room (GR48, Conviron, Winnipeg, MB) located in the controlled environment facilities of the College of Agriculture and Bioresources at the University of Saskatchewan. Distilled water was added to pots as needed, and plants fertilized weekly with 100 mL of the half strength modified Hoagland's nutrient solution.

#### Collection of roots and extraction of polyphenols

All plants in each pot were gently removed 30 d after transplanting. The intact roots were collected, weighed, and transferred into 50 mL tubes containing 10 mL 80% MeOH. Polyphenols that had been released from root tissues were extracted by soaking roots in 80% MeOH for 72 h. The tubes were stored at −20°C until further use. After 3 d, the tube was thawed at room temperature, vortexed, and 1.5 mL was transferred into a 2 mL centrifuge tube, which was centrifuged for 5 min at 12,000 rpm. Then 1 mL of the supernatant was removed and dried using a benchtop centrifugal vacuum concentrator (Labconco, Kansas City, Mo) for approximately 2 h and immediately reconstituted in 200 μL of the recon solution (90:10 H_2_O:MeOH v/v) to reconstitute the extract.

### Composition of polyphenols in root tissues of healthy and diseased lentil plants

#### Experimental design and plant growth conditions

A factorial pot experiment with four lentil genotypes and two infection levels was conducted in a controlled temperature environment. Seeds of the lentil genotypes ZT-4, CDC Maxim, Eston, and *L. ervoides* L01-827A were scarified, surface sterilized, and germinated as described for experiment 1. Eight germinated seeds of each genotype were transplanted into 10 cm plastic pots filled with peat and vermiculite-based media, Sunshine Mix 3® (Sun Gro Horticulture Canada Ltd.). The planting holes in the media were treated with a peat-based *R. leguminosarum* inoculant (Nitragin Nitrastick GC®, Nitragin Inc., Brookfield, WI, USA). The pots were arranged in four replicates. Plants were grown under 22/16°C d/night temperatures and 16/8 h d/night length for 30 d in the growth room (GR48, Conviron, Winnipeg, MB). Distilled water was added to pots as needed, and plants were fertilized weekly with 100 mL of the half strength modified Hoagland's nutrient solution. Roots of the infected plants were inoculated with zoospores of *A. euteiches*. The experiment was repeated two times.

#### Preparation of inoculum of *Aphanomyces euteiches* and root infection

Zoospores of *A. euteiches* were produced following a protocol developed in the Plant Pathology Laboratory, University of Saskatchewan, Canada (Banniza, pers. comm.). After counting the zoospores using a haemocytometer, the final concentration was adjusted to 5 × 10^3^ zoospores mL^−1^ with sterile deionized water. Ten days after planting, a 5-mL aliquot of the zoospore suspension was injected into the soil near the base of the lentil plants.

#### Collection of roots and extraction of root polyphenols

Fourteen days after inoculation, the plants were uprooted and the adhering media (Profile® Greens Grade™, Winnipeg, MB) were removed from the roots by soaking the roots in the tap water. The roots were then visually evaluated for infection on the basis of discoloration and root decay. The roots were stored at −80°C until use.

Polyphenols were extracted from roots of healthy and diseased plants using a procedure similar to that of Mirali et al. (2014) with a few modifications. One hundred fifty milligram of fresh root tissue was placed in 2 mL Sarstedt microtube and then freeze dried over 24 h. The freeze-dried samples were weighed, and then one ¼- inch ceramic bead (MP Bio, Cat. No. 6540-412) was placed in each tube along with 1 mL of an extraction solvent containing 70:30 v/v acetone:water plus internal standards. The root tissues were pulverized using a Mini-Beadbeater-16 (BioSpec Products, Inc. US) for 2 × 2.5 min followed by shaking using an Eppendorf mixer for 1 h at 1,400 rpm and 23°C. After centrifuging the tubes at 12,000 rpm for 5 min, 500 μL of the supernatant was pipetted into 1.5 mL Eppendorf tubes and centrifuged again at 12,000 rpm for 5 min. From the Eppendorf tube, 200 μL of the supernatant was pipetted into a new 1.5 mL Eppendorf tube and dried down for approximately 2 h using a benchtop centrifugal vacuum concentrator (Labconco, Kansas City, MO). The supernatant was then reconstituted by adding 200 μL of 90:10 MiliQ-water:MeOH to each tube followed by vortexing for 20 sec and shaking on an Eppendorf mixer for 30 min at 1,400 rpm at 23°C. The tubes were centrifuged at 12,000 rpm for 5 min and then 150 μL of the supernatant was transferred into LC vials and used for LC-MS analysis.

### LC-MS analysis

Polyphenols were purchased from Sigma-Aldrich (Missouri, USA), and Extrasynthese (Genay, France) except for the deuterated compounds, which were purchased from Toronto Research Compounds (Toronto, Canada).

Root tissues and root extracts of the lentil plants were analyzed for polyphenols using a diode-array detector (DAD) for UV-vis detection and a targeted LC-MS method. Since all polyphenols absorb UV-vis, the DAD detector detects all polyphenols in the sample and the MS gives the *m/z* of the ion(s) within each peak. The targeted method (list of polyphenols analyzed is given in Table 1) uses LC-selective reaction monitoring (SRM) and is based on the method initially developed by Mirali et al. (2014), that has been more recently updated (Mirali et al., 2016a; Purves et al., 2016). Note that catechin-^13^C_3_, 4-hydroxybenzoic acid-^13^C_7_, ferulic acid-d_3_, resveratrol 4-hydroxyphenyl-^13^C_6_, vanillin ring-^13^C_3_, and quercetin-d_3_ were used as internal standards. The *m/z* values used for the parent and fragment ions for each of the polyphenols are given in Table 1.

**Table 1:** Polyphenols analyzed in the root extracts and root tissues of cultivated lentil genotypes ZT-4, CDC Maxim, Eston, and *Lens ervoides* L01-827A using liquid chromatography-mass spectrometry.

**Compound**	**Retention time (min)**	**Mode**	**Molecular ion (*m/z*)**	**Fragment ion (*m/z*)**
Gallic acid	2.1	NEG^a^	169	125
Salicin	3.3	NEG	285	123
3,4-dihydroxybenzoic acid	3.5	NEG	153	109
4-aminosalicylic acid	3.5	NEG	152	108
Vanillic acid 4-glucoside	4.1	NEG	329	167
Gallocatechin	4.5	NEG	305	125
4-hydroxybenzoic acid	4.8	NEG	137	93
4-hydroxybenzoic acid-13C7^*^	4.8	NEG	144	99
Delphinidin 3,5-diglucoside	5.5	NEG	625	299
Procyanidin B1	5.8	NEG	577	289
Cyanidin 3,5-diglucoside	6.1	NEG	609	447
Catechin 3-glucoside	6.1	NEG	451	137
Vanillic acid	6.2	NEG	167	108
Epigallocatechin	6.3	NEG	305	125
Catechin	6.4	NEG	289	203
Catechin 13C3^*^	6.4	NEG	292	206
Chlorogenic acid	6.8	NEG	353	191
Caffeic acid	6.8	NEG	179	135
Delphinidin 3-glucoside	6.9	NEG	463	300
Quercetin 3-galactoside	11.0	NEG	463	300
Quercetin 3-glucoside	11.2	NEG	463	300
Procyanidin B2	6.9	NEG	577	289
Malvidin 3,5-diglucoside	7.3	NEG	653	329
Vanillin ring 13C6^*^	7.5	NEG	157	142
Epicatechin	7.8	NEG	289	203
Dihydromyricetin	8.0	NEG	319	193
p-Coumaric acid	8.4	NEG	163	119
Kaempferol 3-rhamnoside	14.6	NEG	431	285
Kaempferol dirutinoside	8.7	NEG	901	755
Cyanidin 3-rhamnoside	8.7	NEG	431	285
Malvidin 3-galactoside	8.7	NEG	491	313
Malvidin 3-glucoside	9.1	NEG	491	313
3-hydroxycinnamic acid	9.3	NEG	163	119
3-hydroxy-4-methoxycinnamic acid	9.6	NEG	193	134
Ferulic acid	9.6	NEG	193	134
Ferulic acid-d3^*^	9.6	NEG	196	134
Kaempferol 3-rutinoside 4′-glucoside	9.6	NEG	755	593
Quercetin 3,4′-diglucoside	9.7	NEG	625	463
Luteolin 8′-glucoside	9.7	NEG	447	327
Kaempferol 3-robinoside 7-rhamnoside	10.2	NEG	739	593
Taxifolin	10.2	NEG	303	125
Resveratrol 3-glucoside	10.5	NEG	389	227
Luteolin 3′,7-diglucoside	10.5	NEG	609	285
Naringenin 7-rutinoside	11.6	NEG	597	271
Epicatechin gallate	10.6	NEG	441	169
Myricetin 3-rhamnoside	10.8	NEG	463	316
Quercetin 3-rutinoside	10.8	NEG	609	300
Luteolin 7-rutinoside	11.4	NEG	593	285
Luteolin 7-glucoside	11.6	NEG	447	285
Kaempferol 3-galactoside	11.7	NEG	447	255
Kaempferol 3-rutinoside	11.8	NEG	593	285
Dihydrokaempferol	11.9	NEG	287	125
Kaempferol 3-glucoside	12.1	NEG	447	285
Quercetin 3-rhamnoside	12.2	NEG	447	300
Hesperetin 7-rutinoside	12.3	NEG	609	301
Kaempferol 7-neohesperidoside	12.3	NEG	593	285
Apigenin 7-rutinoside	12.4	NEG	577	269
Kaempferol 7-glucoside	12.4	NEG	447	285
Apigenin 7-neohesperidoside	12.6	NEG	577	269
Apigenin 7-glucoside	12.8	NEG	431	268
Diosmetin 7-rutinoside	12.8	NEG	607	299
Quercetin 4′-glucoside	13.0	NEG	463	301
Myricetin	13.2	NEG	317	151
Luteolin 4′-glucoside	13.3	NEG	447	285
Resveratrol 4-hydroxyphenyl 13C6^*^	13.5	NEG	233	149
Resveratrol	13.5	NEG	227	143
4-hydroxy-6-methylcoumarin	13.8	NEG	175	131
Eriodicytol	14.6	NEG	287	151
Quercetin	15.4	NEG	301	151
Quercetin-d3^*^	15.4	NEG	304	151
Luteolin	15.8	NEG	285	133
Naringenin	16.6	NEG	271	151
Genistein	16.7	NEG	269	133
Hesperetin	17.2	NEG	301	164
Phloretin	17.5	NEG	273	167
Kaempferol	17.5	NEG	285	187
Apigenin	17.6	NEG	269	117
Isorhamnetin	17.8	NEG	315	300
Diosmetin	17.9	NEG	299	284
Flavone	20.0	POS^b^	223	77
5,7-dimethoxyflavone	20.8	POS	283	239
Flavanone	21.8	POS	225	121
Xanthohumol	25.1	POS	355	179

* *Internal standard*.

a *Negative ionization mode*.

b *Positive ionization mode*.

Previous chromatographic conditions optimized by Mirali et al. (2016a) were applied with some modifications. An Agilent 1290 UPLC (Agilent Technologies, Santa Clara, CA) equipped with an auto sampler (G4226A), a binary pump (G4220), a thermostatted column compartment (G1316), and a diode array detector (G4212) was used. Compounds were separated using a Phenomenex (Torrance, CA) core-shell Kinetex pentafluorophenyl (PFP) column (100 mm × 2.1 mm, 2.6 μm particle size). The mobile phase employed a 30 min binary gradient shown in Table 2; solvent A consisted of H_2_O: CH_2_O_2_ 99:1, and solvent B was ACN:H2O: CH_2_O_2_ 90:9:1 (v/v). The injection volume was 5 μL and the flow rate was 0.35 mL/min. Polyphenols exiting the column were detected by the DAD and then subsequently with a Thermo Fisher TSQ Vantage triple quadrupole mass spectrometer equipped with a heated electrospray ionization (HESI) interface.

**Table 2:** Binary gradient used in this study.

**Time (min)**	**A%**	**B%**	**Flow (mL/min)**
0	99	1	0.35
1	99	1	0.35
21	59	41	0.35
24	40	60	0.35
24.1	20	80	0.35
26	20	80	0.35
26.1	99	1	0.35
30	99	1	0.35

*Solvent A consists of water: formic acid (99;1, v/v), and solvent B consists of water: acetonitrile: formic acid (9:90:1, v/v/v)*.

Data were processed by comparing peak areas of polyphenols normalized to the peak area of an internal standard (IS) using Thermo Xcalibur 2.2 software. Each compound (Table 1) was quantified using a 4-point calibration curve that was obtained using serial dilutions. Kaempferol dirutinoside and catechin-3-glucoside were not commercially available, however, their presence has been reported in lentil seeds, and they were examined here based on previous reports (Aguilera et al., 2010; Mirali et al., 2016a). The standard curves of kaempferol 3-rutinoside and catechin were used to estimate the quantities of these compounds, respectively. Concentration values were converted to ng/g based on dry sample weight.

### Bioassay of the effect of polyphenols on mycelial growth of *Aphanomyces euteiches*

The polyphenols found in the root tissues and root extracts were evaluated for their effect on mycelial growth of the pathogen *A. euteiches* (AE1) isolated from Saskatchewan soils. Commercially available polyphenols were used to prepare the stock solutions. A 1 mm well was created in the center of 5% corn meal agar (CMA) plates. An aliquot of 20 μL of 1 g L^−1^ of each polyphenol was added to the well.

Control plates received 20 μL of sterilized deionized water and a 1 mm plug of *A. euteiches* was cut from the margins of an actively growing culture plate and placed in the well. Inoculated plates were incubated in the dark at 22°C and radial growth was measured daily for up to 5 d. Percentage inhibition or stimulation of radial growth was calculated using the formula:

$$\begin{array}{l} \%\text{age growth inhibition}/\text{stimulation}=\left(\text{C}-\text{T}\right)/\text{C}\times 100 \end{array}$$

Where C = growth in the control plate and T = growth in the treated plate (Dickinson and Skidmore, 1976). The experiment was repeated three times with three replicated plates per treatment.

Polyphenols found to be active against the pathogen *A. euteiches* were selected for further examination of their minimum inhibitory concentration. Using the same protocol described above, three concentrations of the selected polyphenols were examined for their effect on radial growth of the pathogen. Each concentration included a 10 times dilution level. We used a wide range of concentrations for each polyphenol to detect the possible inhibitory effects. The experiment was repeated three times with three replicated plates per treatment.

### Statistical analysis

Analysis of variance (ANOVA) was performed to test the significance of the effect of genotype, treatments and the interactions between genotype and treatment on the concentration of polyphenols in roots tissues. The least significant difference (LSD) test was used to determine differences in the concentration of the compounds among genotypes and between the treatments. LSD was also used to test differences among genotypes in the concentration of the compounds in the root extract. A *p*-value of 0.05 was used as the threshold below which the null hypothesis was rejected. Comparisons of mean values and principal component analysis (PCA) were completed using R package (R, v.3.3.2) (www.r-project.org). A Dunnett's test was used to compare the *A. euteiches* mycelial inhibition of each polyphenol/concentration with control (*P* < 0.05).

## Results

**Figure 1:**
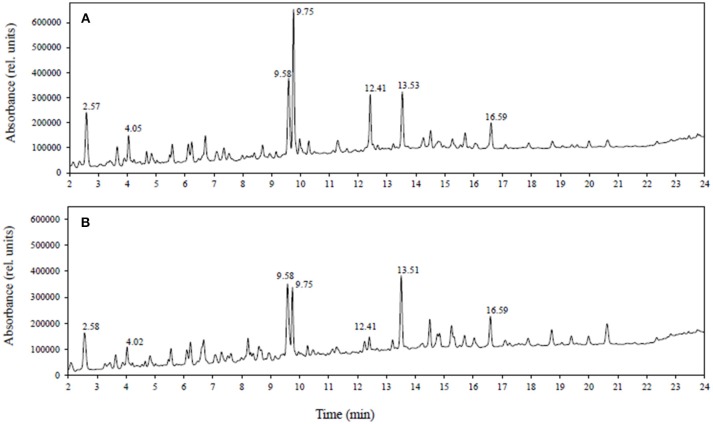
LC-DAD spectra (UV-vis: 250–600 nm) obtained from tissue extracts of **(A)** healthy roots and **(B)** diseased roots of lentil genotype ZT-4.

Since all polyphenols absorb in the UV-vis region, the combination of LC-DAD with LC-MS is a powerful approach for investigating polyphenols. Figure 1 shows a comparison of LC-DAD spectra (UV-vis 250–600 nm) obtained using extracts of (A) healthy roots and (B) diseased root tissues of lentil genotype ZT-4. The figure shows that the most significant changes in the spectra are observed at 9.75 and 12.41 min, with less significant changes being observed at other retention times. The compound at 9.75 min has an *m/z* of 755 (negative mode MS) and no standard has this *m/z* and retention time in our targeted LC-SRM method. This compound was observed to have a similar product ion spectrum compared with kaempferol 3-O-rutinoside-4′-glucoside (*m/z* 755, tr = 9.6 min included in the targeted method) and therefore is consistent with being a structural isomer of kaempferol 3-O-rutinoside-4′-glucoside. Similarly, the compound at 12.41 min has an *m/z* of 901 (negative mode) and no standard has this *m/z* and retention time in our targeted method. This unknown compound was observed to have many of the same product ions as kaempferol dirutinoside (*m/z* 901, tr = 8.69 min, included in the targeted method) and therefore is consistent with being a structural isomer of kaempferol dirutinoside. Thus, the UV-vis spectra not only detected large changes in the profile but also the presence of polyphenols not included in the targeted SRM method. For this study, we are focusing on quantifying polyphenols already present in our library.

### Biochemical analysis of root extracts

**Figure 2:**
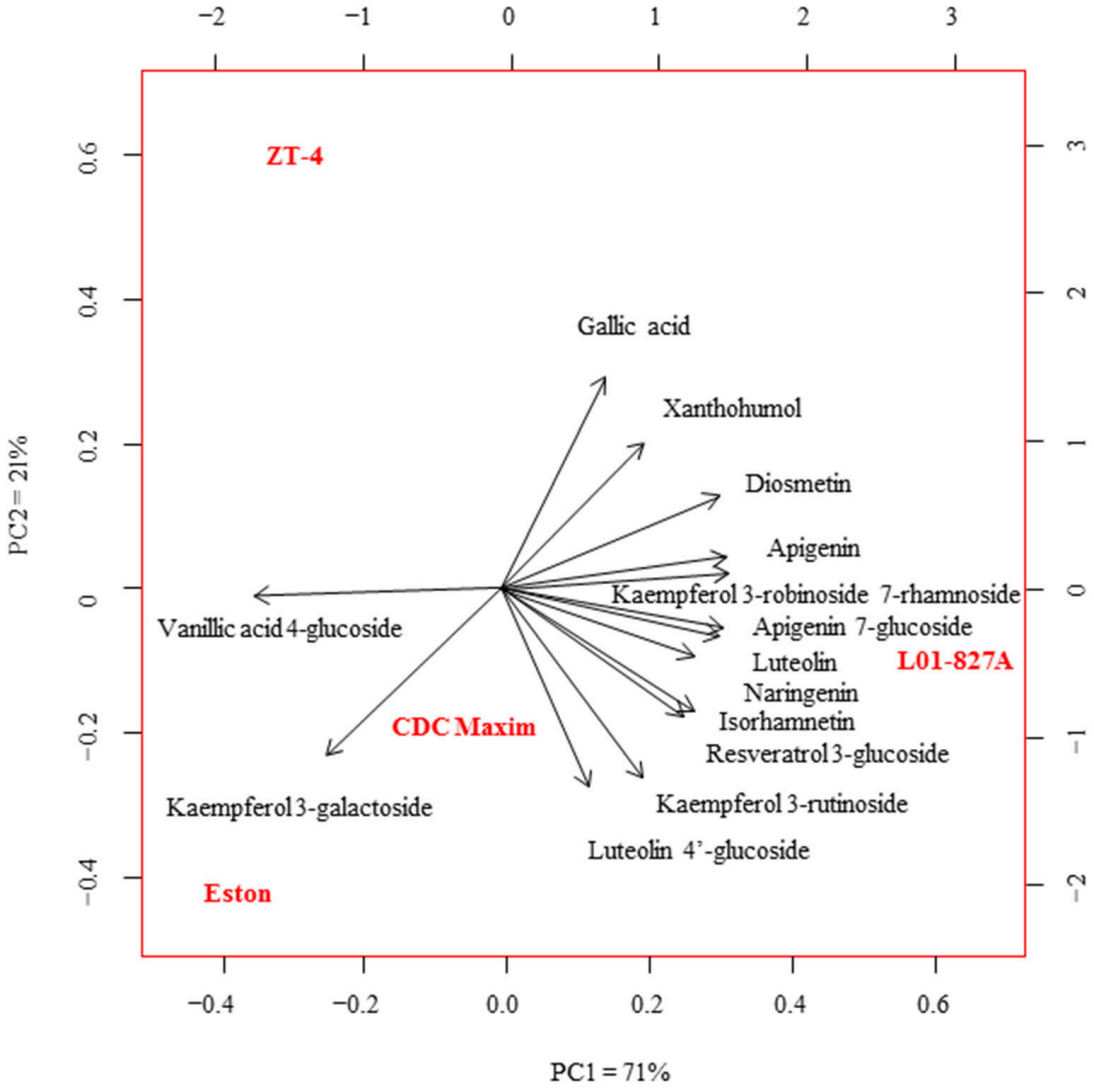
Principal component analysis of the concentration of phenolic compounds in the root extracts of cultivated lentil genotypes ZT-4, CDC Maxim and Eston and *Lens ervoides* L01-827A using liquid chromatography-mass spectrometry (*n* = *4*). Phenolic compounds were extracted from root extracts using 80% MeOH, and analyzed using LC-SRM.

Fourteen polyphenols detected by LC-SRM were common to the root extracts of all 4 genotypes of lentil. Variation in the level of these polyphenols among the 4 genotypes of lentil was shown in Figure 2. *Lens ervoides* L01-827A had elevated amounts of most of the polyphenols in its root extract (Table 3).

**Table 3:** Concentration of phenolic compounds (ng/g root) detected in the root extracts of cultivated lentil genotypes ZT-4, CDC Maxim and Eston and *Lens ervoides* L01-827A using liquid chromatography-mass spectrometry.

**Compound**	**Genotype**			
	**ZT-4**	**Eston**	**CDC Maxim**	**L01-827A**
Vanillic acid 4-glucoside	5886 ± 2123^1^ a	7166 ± 2947 a^2^	3433 ± 1782 ab	2302 ± 1143 b
Diosmetin	436 ± 89 b	180 ± 61 c	310 ± 204 bc	888 ± 72 a
Apigenin	43.7 ± 13.4 b	33.5 ± 20.6 b	56.2 ± 29.6 ab	87.6 ± 27.8 a
Naringenin	24.4 ± 3.5 b	29.3 ± 9.4 b	24.4 ± 12.4 b	62.1 ± 15.4 a
Kaempferol 3-robinoside 7-rhamnoside	2.08 ± 0.98 b	1.74 ± 1.19 b	2.46 ± 2.24 b	9.85 ± 4.36 a
Gallic acid	1.72 ± 0.01 a	1.11 ± 0.73 a	1.20 ± 1.04 a	1.61 ± 0.52 a
Kaempferol 3-galactoside	1.58 ± 1.17 ab	3.67 ± 1.52 a	3.48 ± 3.31 ab	1.05 ± 0.75 b
Apigenin 7-glucoside	0.68 ± 0.32 b	0.63 ± 0.44 b	1.02 ± 0.83 ab	2.88 ± 2.36 a
Luteolin	0.64 ± 0.18 b	0.20 ± 0.15 b	1.33 ± 1.19 b	15.57 ± 0.62 a
Kaempferol 3-rutinoside	0.22 ± 0.2 a	0.28 ± 0.1 a	0.29 ± 0.2 a	0.37 ± 0.2 a
Isorhamnetin	0.23 ± 0.09 b	0.35 ± 0.3 ab	0.31 ± 0.2 b	0.69 ± 0.2 a
Xanthohumol	0.03 ± 0.03 a	0.02 ± 0.02 a	0.03 ± 0.03 a	0.03 ± 0.03 a
Luteolin 4′-glucoside	0.02 ± 0.02 b	0.07 ± 0.07 ab	0.11 ± 0.06 ab	0.15 ± 0.08 a
Resveratrol 3-glucoside	0.05 ± 0.05 b	0.09 ± 0.09 b	0.37 ± 0.3 ab	0.86 ± 0.7 a

1 *Standard deviation*.

2 *Significant differences were detected using a Least Significant Difference (LSD) test (n = 4) (p < 0.05). Different letters indicate significant differences between means*.

### Biochemical analysis of healthy and diseased root tissues

**Figure 3:**
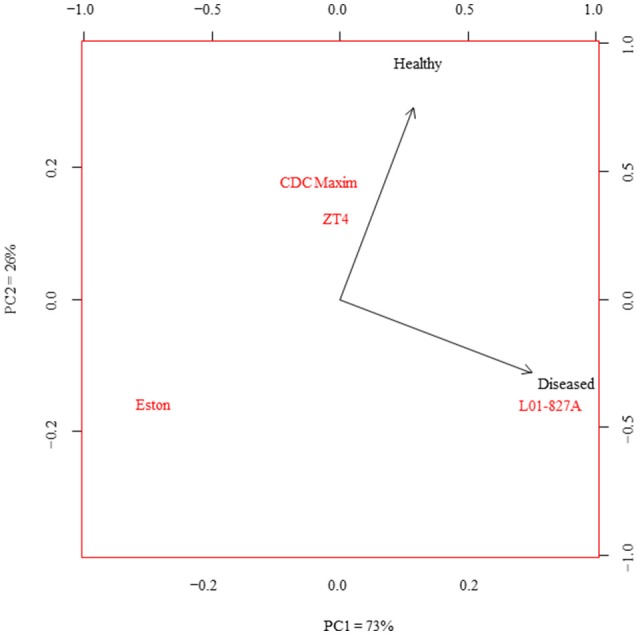
Principal component analysis of the concentration of phenolic compounds in the healthy and diseased root tissues of cultivated lentil genotypes ZT-4, CDC Maxim and Eston and *Lens ervoides* L01-827A using liquid chromatography-mass spectrometry (*n* = *4*). Phenolic compounds were extracted from root tissues, and analyzed using LC-SRM.

The LC-SRM analysis detected 17 polyphenols in the root tissues of all 4 lentil genotypes. The effect of genotype, infection and the interactions between genotype and infection on the concentration of each of these polyphenols was described in Table 4. Concentrations of some of the polyphenols varied among the genotypes in healthy plants, whereas in diseased plants, the concentration of most of the polyphenols varied among the genotypes (Table 5). Concentration of several compounds varied between diseased and healthy root tissues of the same genotype. The variation observed in the concentration of polyphenols between healthy and diseased roots was larger in *L. ervoides* L01-827A whereas it was smaller in ZT-4 and CDC Maxim (Figure 3). The concentration of apigenin, kaempferol, and naringenin was higher in diseased root tissues for all four lentil genotypes (Table S1). Diosmetin, hesperetin 7-rutinoside, kaempferol 3-galactoside, kaempferol 3-rutinoside, naringenin 7-rutinoside, and vanillic acid were only significantly more abundant in diseased roots of *L. ervoides* L01-827A (Table S1). Coumaric acid was only detected in diseased roots of *L. ervoides* L01-827A and Eston. 3,4-dihydroxybenzoic acid was detected in diseased roots of all and in healthy roots of *L. ervoides* L01-827A (Table S2).

**Table 4:** Statistical significance of the effect^a^ of genotype and infection on the composition of some phenolic compounds in the root tissues of cultivated lentil genotypes ZT-4, CDC Maxim, Eston, and *Lens ervoides* L01-827A determined by liquid chromatography-mass spectrometry (*n* = *4*).

**Compound**	**Genotype**	**Infection**	**Genotype × Infection**
Apigenin	0.01	<0.0001	<0.0001
Apigenin 7-glucoside	0.01	0.002	0.04
Dihydrokaempferol	0.51	0.008	0.88
Diosmetin	<0.0001	<0.0001	0.003
Eriodictyol	0.39	0.001	0.25
Hesperetin 7-rutinoside	0.04	0.74	0.001
Isorhamnetin	0.91	0.008	0.92
Kaempferol 3 galactoside	0.39	0.03	0.23
Kaempferol 3-robinoside 7-rhamnoside	0.001	0.006	0.06
Kaempferol 3-rutinoside	0.02	0.04	0.2
Kaempferol dirutinoside	0.001	0.005	0.003
Kaempferol	0.94	<0.0001	0.97
Luteolin	0.03	0.001	0.22
Naringenin 7-rutinoside	0.54	0.48	0.009
Naringenin	0.01	<0.0001	0.001
Vanillic acid 4-glucoside	0.14	0.03	0.28
Vanillic acid	0.09	<0.0001	0.10

a *Significant effects were detected using analysis of variance (ANOVA) (n = 4)*.

**Table 5:** Concentration of phenolic compounds (μg g^−1^ root) detected in the root tissues of cultivated lentil genotypes ZT-4, CDC Maxim and Eston and *Lens ervoides* L01-827A using liquid chromatography-mass spectrometry.

**Compound**	**Treatment**	**Genotype**			
		**ZT-4**	**Eston**	**CDC Maxim**	**L01-827A**
Apigenin	Healthy	1.02 a^1^	0.44 a	0.21 a	0.77 a
	Infected	3.19 b	1.99 b	1.67 b	6.67 a
Apigenin 7-glucoside	Healthy	0.06 a	0.01 a	0.002 a	0.07 a
	Infected	0.07 b	0.12 b	0.05 b	0.32 a
Dihydrokaempferol	Healthy	0.34 a	0.04 a	0.03 a	0.11 a
	Infected	0.69 a	0.21 a	0.35 a	0.75 a
Diosmetin	Healthy	24.3 b	25.5 b	28.6 ab	39.9 a
	Infected	34.4 b	36.9 b	43.6 b	100.7 a
Eriodictyol	Healthy	0.03 a	0.03 a	0.02 a	0.03 a
	Infected	0.05 a	0.05 a	0.05 a	0.08 a
Hesperetin 7-runtinoside	Healthy	0.41 a	0.16 a	0.15 a	0.14 a
	Infected	0.17 b	0.20 b	0.15 b	0.42 a
Isorhamnetin	Healthy	0.61 a	0.44 a	0.27 a	0.76 a
	Infected	1.37 a	1.85 a	1.43 a	1.73 a
Kaempferol 3-galactoside	Healthy	0.20 a	0.12 a	0.09 a	0.09 a
	Infected	0.17 a	0.32 a	0.13 a	0.28 a
Kaempferol 3-robinoside 7-rhamnoside	Healthy	0.99 b	0.68 b	0.74 b	3.28 a
	Infected	1.47 bc	4.73 ab	0.64 c	7.72 a
Kaempferol 3-rutinoside	Healthy	0.19 a	0.15 a	0.03 a	0.11 a
	Infected	0.11 b	0.24 ab	0.04 b	0.41 a
Kaempferol dirutinoside	Healthy	2.21 b	7.04 b	1.73 b	26.9 a
	Infected	3.88 b	78.9 a	0.75 b	42.8 ab
Kaempferol	Healthy	0.39 a	0.46 a	0.22 a	0.18 a
	Infected	4.80 a	4.15 a	3.56 a	4.57 a
Luteolin	Healthy	1.05 a	0.98 a	0.15 a	1.13 a
	Infected	1.86 b	4.40 a	0.86 b	2.86 ab
Naringenin 7-rutinoside	Healthy	0.09 a	0.04 ab	0.05 ab	0.03 b
	Infected	0.04 b	0.04 b	0.05 b	0.08 a
Naringenin	Healthy	0.29 a	0.07 b	0.08 ab	0.11 ab
	Infected	0.66 b	0.57 b	0.85 b	1.85 a
Vanillic acid 4-glucoside	Healthy	32.9 a	32.2 a	21.8 a	21.4 a
	Infected	45.3 ab	94.5 a	35.5 b	41.1 ab
Vanillic acid^2^	Healthy	20.7 a	13.9 a	21.5 a	18.1 a
	Infected	35.8 ab	26.0 b	34.1 ab	54.5 a

1 *Significant differences were detected using a Least Significant Difference (LSD) test (n = 4) (p < 0.05). Different letters indicate significant differences between means*.

2 *Due to a co-eluting interference in the SRM channel, the value of vanillic acid was estimated by LC-UV*.

### *In vitro* assay testing the effect of polyphenols on mycelial growth of *Aphanomyces euteiches*

Seven polyphenols including vanillic acid, 4-hydroxybenzoic acid, 3,4-dihydroxybenzoic acid, 4-aminosalicylic acid, coumaric acid, delphinidin 3-glucoside and phloretin inhibited the mycelial growth of *A. euteiches* at 1 mg mL^−1^ *in vitro* (*p* < 0.0001). Coumaric acid and phloretin also inhibited *A. euteiches* at 0.1 g L^−1^ (10x dilution). No inhibition was observed for other compounds at 0.1 g L^−1^ (10x dilution), or at lower concentrations of any of these polyphenols (Table 6).

**Table 6:** Inhibitory effect of bioactive polyphenols^a^ detected in lentil root tissues on the *in vitro*^*b*^ radial growth of *Aphanomyces euteiches*^*c*^.

**Polyphenol**	**Concentration**		
	**1 g L**^−1^ **(%)**	**0.1 g L**^−1^ **(%)**	**0.05 g L**^−1^ **(%)**
4-hydroxybenzoic acid	100^***d,e^	10.50^nsf^	2.60^ns^
4-aminosalicylic acid	100^***^	6.60^ns^	5.60^ns^
Vanillic acid	100^***^	8.50^ns^	4.60^ns^
Coumaric acid	100^***^	44.90^*^	12.50^ns^
3,4-dihydroxybenzoic acid	100^***^	12.50^ns^	11.50^ns^
Phloretin	61.70^*^	31.10^*^	13.40^ns^
Delphinidin 3-glucoside	21.50^*^	14.50^ns^	11.40^ns^

a *20 μL of phenolic compound was applied*.

b *Corn meal agar (CMA) medium (5%)*.

c *Mycelial growth was measured 5 d after inoculation*.

d *Values represent percent inhibition of radial growth of Aphanomyces euteiches compared to control*.

e Significant differences between treatments and control were detected using Dunnett's test (n = 3) (

*** *p < 0.001*,

* *p < 0.05)*.

f *Not significant*.

## Discussion

We used semi-quantitative mass spectrometry to investigate genotypic variations within the composition of polyphenols that naturally exist in the root tissues and root extracts of lentil. We analyzed a wide range of polyphenols and demonstrated that the qualitative and quantitative composition of polyphenols not only differs among the lentil genotypes, but also between the healthy and *A. euteiches* infected roots.

The higher concentration of polyphenols in the root extracts of the *L. ervoides* genotype (tertiary gene pool) compared to the cultivated *L. culinaris* genotypes (primary gene pool) could be an evolutionary divergence or a consequence of selection for larger plants and seeds during the domestication process (Alo et al., 2011; Wong et al., 2015). It was shown that the selection of new cultivars for specific traits or under optimal growth conditions may inadvertently lead to the loss of specific genes and phytochemicals (Hättenschwiler and Vitousek, 2000; Dixon, 2001) including those involved in the synthesis of polyphenols.

Lentil genotype ZT-4 is characterized by the expression of the single recessive gene, *tan*, that affects the color and thickness of the seed coat (Vaillancourt et al., 1986). The seed coats of low-tannin lentils lack many of the polyphenols found in normal seed coats, causing them to be thinner and more fragile compared to the seed coat of normal genotypes (Matus et al., 1993; Mirali et al., 2016a). This study shows that the *tan* gene is also expressed in the root tissues and results in a lower concentration of most of the polyphenols.

Variations observed in the levels of specific polyphenols between healthy and diseased root tissues may be due to defensive responses through the inactivation of microbial enzymes, reinforcement of plant structural components or creation of strategic barriers at points of entry (Sylvia and Sinclair, 1983; Beckman, 2000; Vermerris and Nicholson, 2008). In this study, the elevated concentration of apigenin, kaempferol, and naringenin in the diseased roots of all four lentil genotypes (Table S1) may suggest that these three polyphenols are associated with a general response against *A. euteiches*. Support for this hypothesis can be found in previous studies in which naringenin and apigenin were shown to have inhibitory effects on the bacterial pathogens and nematodes (Baidez et al., 2006; Treutter, 2006). Since such inhibitory effect was not detected against *A. euteiches* in our bioassay, these polyphenols might have other potential roles in a response against *A. euteiches*. Similar levels of several compounds in healthy and diseased roots of the low-tannin genotype ZT-4 may suggest that the expression of the gene *tan* is one of the factors influencing the composition of polyphenols in responses to phytopathogens. The differences observed in the concentration of polyphenols between healthy and diseased roots in different genotypes (Tables 3, 4) may indicate that the synthesis of defensive metabolites is genotype dependent. The variation was larger in *L. ervoides* L01-827A (Figure 3), suggesting that this wild genotype might have a wider capacity for a defensive response to plant pathogens.

In this study, the lentil genotypes were treated by the endosymbiont *R. leguminosarum*. It is notable that some polyphenols act as chemoattractants for rhizobium, improving the root colonization (Fisher and Long, 1992; Begum et al., 2001; Cooper, 2004) and, rhizobium may aid the plant to resist against pathogen by mediating the polyphenolic content of root tissues (Mishra et al., 2006). However, rhizobium may interact differently with the lentil genotypes resulting in different degrees of root colonization, and different responses to plant pathogens.

Certain phytochemicals were shown to attract the zoospores or inhibit the hyphal growth of *A. euteiches* (Yokosawa et al., 1986; Smolinska et al., 1997; Cannesan et al., 2012). In our study, the first involving lentil, some of the root polyphenols that inhibited the hyphal growth of the *A. euteiches* were more concentrated in specific lentil genotypes. For instance, the concentration of vanillic acid and 3,4-dihydroxybenzoic acid were higher in *L. ervoides* L01-827A which has a marbled gray seed coat. Coumaric acid was detected only in diseased roots of *L. ervoides* L01-827A, and in the green seed coat of the cultivar Eston (Mirali et al., 2016b). Differences in the quantity and quality of the phenylpropanoid metabolites in different genotypes of lentil suggests there could be useful genotypic variation in the level of resistance to *A. euteiches* in lentil. Since the majority of these *A. euteiches* inhibitors were organic acids, the organic acid content of root tissues and extracts appears to be associated with some level of resistance to ARR. Organic acids comprise a major portion of root extracts in most plant species (Neumann and Römheld, 2001; Cawthray, 2003; Sandnes et al., 2005). Organic acids and their derivatives were detected in both root tissue and root extracts of lentil. The observation that some organic acids, including caffeic acid, gallic acid, and 3-hydroxy-4-methoxycinnamic acid, did not affect the hyphal growth of *A. euteiches* suggests that the inhibitory effect on *A. euteiches* is limited to specific organic acids. The concentrations of root polyphenols could be variable in real conditions depending on various factors. However, an accumulative concentration of the bioactive polyphenols could be achieved and be effective to inhibit the root rot pathogen. Polyphenols, particularly those released from roots, are involved in a wide range of agro-ecological processes, and could affect specific ecological niches in the rhizosphere (Aira et al., 2010; Doornbos et al., 2012). The selective release of organic acids and other polyphenols that inhibit *A. euteiches* may improve soil health by reducing the abundance of the pathogen in soil, and the severity of ARR in subsequent susceptible crops in rotation.

To date there is no report of detection of effective resistance to *A. euteiches* in lentil. Among the genotypes used in this study, ZT4 was more susceptible to *A. euteiches* (Knowpulse Report, 2015), while L01-827A and Eston have shown a moderate level of resistance to *A. euteiches* (Banniza, pers. comm.; Saskpulse, 2015). One reason for their better tolerance to *A. euteiches* could be the elevated levels of specific of polyphenols. Such variations could increase the understanding of pathways of biochemical resistance to plant diseases and could be used as a starting point for identification of lentil genotypes that are resistant to root rot diseases in breeding programs.

Our study revealed that genotypic variations for the composition of polyphenols exist in lentil, and that there is a relationship between the composition of polyphenols and tolerance to *A. euteiches*. The *tan* gene in lentil influences the composition of polyphenols in root extracts. A number of studies have described the potential role of polyphenols in providing resistance against root rot diseases in different plant species (Farkas and Kiraaly, 1962; Lagrimini et al., 1993; Daayf et al., 2012; Aksoya et al., 2017). Such studies usually focus on a small number of compounds and thus cannot provide a clear image of the function of polyphenols. Here, we analyzed a wide range of polyphenols in response to root rot disease. Understanding of the role of polyphenols in providing resistance against root rot diseases, and their potential application in breeding programs is still not well defined. In lentil breeding programs, the exploitation of polyphenol content has mainly targeted the seed coats in order to improve seed quality, storage, marketability and nutritional values (Vaillancourt et al., 1986; Mirali et al., 2016b, 2017; Ganesan and Xu, 2017). Further research is required to identify and quantify all the polyphenols that are differentially expressed in uninfected and infected root tissues of lentil species. It is necessary to detect correlations between the concentration of specific polyphenols and other metabolites in the root tissues in order to identify mechanisms of resistance to *A. euteiches*. The induction of resistance in host plants can be influenced by a number of factors, including plant genotype, nutrition and environmental conditions (Walters et al., 2013). Therefore, it is necessary to understand the influence of plant nutrition on the polyphenolic content of roots, since this may override the influence of plant genetics on the composition of polyphenols. We are currently investigating unknown peaks in the UV spectra to identify new compounds of importance for roots that will be added to the polyphenol library in future studies.

## Author contributions

NB grew plants and collected the root materials. NB and RP contributed to LCMS analyses. PP prepared the inoculum and conducted the *in-vitro* plate assay. All authors contributed to experimental design and to preparation of the manuscript.

### Conflict of interest statement

The authors declare that the research was conducted in the absence of any commercial or financial relationships that could be construed as a potential conflict of interest.

## Abbreviations

ARR: Aphanomyces Root Rot
CDC: Crop Development Centre
LC: Liquid Chromatography
MS: Mass Spectrometry
SRM: Single Reaction Monitoring
ZT: Zero Tannin.
