# Supplementary material for: Polyphenolic Composition of Lentil Roots in Response to Infection by Aphanomyces euteiches
Source: Front Plant Sci. 2018 Aug 3;9:1131. doi: 10.3389/fpls.2018.01131 (PMC6085569; doi:10.3389/fpls.2018.01131)
Supplement: Supplementary file 1 [file Table_1.docx]

***Supplementary Material***

**Polyphenolic Composition of Lentils Roots in Response to Infection by *Aphanomyces euteiches***

**Navid Bazghaleh*, Pratibha Prashar, Randy W. Purves, and Albert Vandenberg**

Department of Plant Sciences, University of Saskatchewan, Saskatoon, SK, Canada

***Correspondence:** Navid Bazghaleh: Email: navid.bazghaleh@usask.ca

**Table S1.** Concentration^1^ of phenolic compounds (µg g^-1^ root) detected in the healthy and diseased root tissues of cultivated lentil genotypes ZT-4, CDC Maxim and Eston and Lens ervoides L01-827A using liquid chromatography-mass spectrometry.

| **Compound** | **Genotype** | **Healthy roots** | **Infected roots** |
| --- | --- | --- | --- |
| Apigenin | ZT-4 | 1.02 b^2^ | 3.19 a |
|  | Eston | 0.44 b | 1.99 a |
|  | CDC Maxim | 0.21 b | 1.67 a |
|  | L01-827A | 0.77 b | 6.67 a |
|  |  |  |  |
| Apigenin 7-glucoside | ZT-4 | 0.06 a | 0.07 a |
|  | Eston | 0.01 b | 0.12 a |
|  | CDC Maxim | 0.002 b | 0.05 a |
|  | L01-827A | 0.07 b | 0.32 a |
|  |  |  |  |
| Dihydrokaempferol | ZT-4 | 0.34 a | 0.69 a |
|  | Eston | 0.04 b | 0.21 a |
|  | CDC Maxim | 0.03 b | 0.35 a |
|  | L01-827A | 0.11 b | 0.75 a |
|  |  |  |  |
| Diosmetin | ZT-4 | 24.3 a | 34.4 a |
|  | Eston | 25.5 a | 36.9 a |
|  | CDC Maxim | 28.6 a | 43.6 a |
|  | L01-827A | 39.9 b | 100.7 a |
|  |  |  |  |
| Eriodictyol | ZT-4 | 0.03 a | 0.05 a |
|  | Eston | 0.03 a | 0.05 a |
|  | CDC Maxim | 0.02 b | 0.05 a |
|  | L01-827A | 0.03 b | 0.08 a |
|  |  |  |  |
| Hesperetin 7-runtinoside | ZT-4 | 0.41 a | 0.17 a |
|  | Eston | 0.16 a | 0.20 a |
|  | CDC Maxim | 0.15 a | 0.15 a |
|  | L01-827A | 0.14 b | 0.42 a |
|  |  |  |  |
| Isorhamnetin | ZT-4 | 0.61 a | 1.37 a |
|  | Eston | 0.44 a | 1.85 a |
|  | CDC Maxim | 0.27 b | 1.43 a |
|  | L01-827A | 0.76 b | 1. 73 a |
|  |  |  |  |
| Kaempferol 3-galactoside | ZT-4 | 0.20 a | 0.17 a |
|  | Eston | 0.12 a | 0.32 a |
|  | CDC Maxim | 0.09 a | 0.13 a |
|  | L01-827A | 0.09 b | 0.28 a |
|  |  |  |  |
| Kaempferol 3-robinoside 7-rhamnoside | ZT-4 | 0.99 a | 1.47 a |
|  | Eston | 0.68 b | 4.73 a |
|  | CDC Maxim | 0.74 a | 0.64 a |
|  | L01-827A | 3.28 b | 7.72 a |
|  |  |  |  |
| Kaempferol 3-rutinoside | ZT-4 | 0.19 a | 0.11 a |
|  | Eston | 0.15 a | 0.24 a |
|  | CDC Maxim | 0.03 a | 0.04 a |
|  | L01-827A | 0.11 b | 0.41 a |
|  |  |  |  |
| Kaempferol dirutinoside | ZT-4 | 2.21 a | 3.88 a |
|  | Eston | 7.04 b | 78.9 a |
|  | CDC Maxim | 1.73 a | 0.75 a |
|  | L01-827A | 26.9 a | 42.7 a |
|  |  |  |  |
| Kaempferol | ZT-4 | 0.39 b | 4.80 a |
|  | Eston | 0.005 b | 4.15 a |
|  | CDC Maxim | 0.004 b | 3.56 a |
|  | L01-827A | 0.18 b | 4.57 a |
|  |  |  |  |
| Luteolin | ZT-4 | 1.05 a | 1.86 a |
|  | Eston | 0.98 b | 4.40 a |
|  | CDC Maxim | 0.15 b | 0.86 a |
|  | L01-827A | 1.13 b | 2.86 a |
|  |  |  |  |
| Naringenin 7-rutinoside | ZT-4 | 0.09 a | 0.04 a |
|  | Eston | 0.04 a | 0.04 a |
|  | CDC Maxim | 0.05 a | 0.05 a |
|  | L01-827A | 0.03 b | 0.08 a |
|  |  |  |  |
| Naringenin | ZT-4 | 0.29 b | 0.66 a |
|  | Eston | 0.07 b | 0.57 a |
|  | CDC Maxim | 0.08 b | 0.85 a |
|  | L01-827A | 0.11 b | 1.85 a |
|  |  |  |  |
| Vanillic acid 4-glucoside | ZT-4 | 32.9 a | 45.3 a |
|  | Eston | 32.2 b | 94.5 a |
|  | CDC Maxim | 21.8 a | 35.5 a |
|  | L01-827A | 21.4 a | 41.1 a |
|  |  |  |  |
| Vanillic acid^3^ | ZT-4 | 20.7 a | 35.8 a |
|  | Eston | 13.9 a | 26.0 a |
|  | CDC Maxim | 21.5 a | 34.1 a |
|  | L01-827A | 18.1 b | 54.5 a |

^1^ Comparison for each compound was made between the healthy and infected roots of each genotype.

^2^ Significant differences were detected using a Least Significant Difference (LSD) test (*n=4*) (*p<0.05*).

^3^ Due to a co-eluting interference in the SRM channel, the value of vanillic acid was estimated by LC-UV.
