# Supplementary material for: Polyphenolic Composition of Lentil Roots in Response to Infection by Aphanomyces euteiches
Source: Front Plant Sci. 2018 Aug 3;9:1131. doi: 10.3389/fpls.2018.01131 (PMC6085569; doi:10.3389/fpls.2018.01131)
Supplement: Supplementary file 2 [file Table_2.DOCX]

***Supplementary Material***

**Polyphenolic Composition of Lentils Roots in Response to Infection by *Aphanomyces euteiches***

**Navid Bazghaleh*, Pratibha Prashar, Randy W. Purves, and Albert Vandenberg**

Department of Plant Sciences, University of Saskatchewan, Saskatoon, SK, Canada

***Correspondence:** Navid Bazghaleh: Email: navid.bazghaleh@usask.ca

**Table S2.** Concentration of phenolic compounds (µg g^-1^ root) detected in the root tissues of specific lentil genotypes including ZT-4, CDC Maxim and Eston and *Lens ervoides* L01-827A using liquid chromatography-mass spectrometry.

| **Compound** | **Treatment** |  | **Genotype** | |  |
| --- | --- | --- | --- | --- | --- |
|  |  | **ZT4** | **Eston** | **CDC Maxim** | **L01-827A** |
| Coumaric acid | Healthy | ND | ND | ND | ND |
|  | Infected | ND | 6.67 | ND | 6.82 |
| \|  \|  \|  \|  \|  \|  \| \| --- \| --- \| --- \| --- \| --- \| --- \| | \|  \|  \|  \|  \|  \|  \| \| --- \| --- \| --- \| --- \| --- \| --- \| | \|  \|  \|  \|  \|  \|  \| \| --- \| --- \| --- \| --- \| --- \| --- \| | \|  \|  \|  \|  \|  \|  \| \| --- \| --- \| --- \| --- \| --- \| --- \| | \|  \|  \|  \|  \|  \|  \| \| --- \| --- \| --- \| --- \| --- \| --- \| | \|  \|  \|  \|  \|  \|  \| \| --- \| --- \| --- \| --- \| --- \| --- \| |
| 3,4-dihydroxy benzoic acid | Healthy | ND | ND | ND | 0.02 |
|  | Infected | 0.07 | 0.06 | 0.07 | 0.09 |

^1^ND = Not Detected
